# Differentiation-Associated Genes Regulated by c-Jun and Decreased in the Progression of Esophageal Squamous Cell Carcinoma

**DOI:** 10.1371/journal.pone.0096610

**Published:** 2014-05-05

**Authors:** Aiping Luo, Xinfeng Yu, Guichang Li, Gang Ma, Hongyan Chen, Fang Ding, Yi Li, Zhihua Liu

**Affiliations:** 1 State Key Lab of Molecular Oncology, Cancer Institute & Hospital, Chinese Academy of Medical Sciences, Beijing, China; 2 Department of Pharmacology, School of Chemical Biology & Pharmaceutical Sciences, Capital Medical University, Beijing, China; 3 Department of Media and Biology Control, Institute for Communicable Disease Control and Prevention, Chinese Center for Disease Control and Prevention, Beijing, China; The University of Hong Kong, Hong Kong

## Abstract

Transcription factor c-Jun plays a key role in controlling epithelium cell proliferation, apoptosis and differentiation. However, molecular mechanism and biological functions of c-Jun in squamous differentiation and the progression of esophageal squamous cell carcinoma (ESCC) remain elusive. In this study, we found that c-Jun bound directly to the promoter region, and activated the transcription of differentiation-associated genes including cystatin A, involucrin and SPRR3 *in vivo*. Ectopic expression of c-Jun enhanced SPRR3 transactivation in KYSE450 cells. Conversely, TAM67, a dominant negative mutant of c-Jun, inhibited SPRR3 transactivation. c-Jun increased expression of SPPR3 mainly via a PKC/JNK pathway in response to TPA in KYSE450 cells. Furthermore, c-Jun was remarkably reduced in esophageal cancer. Interestingly, cystatin A, involucrin and SPRR3 were significantly downregulated as well, and associated with differentiation grade. Expression of c-Jun was correlated with the expression of these genes in normal epithelium and ESCC. Importantly, the expression of these genes was remarkably decreased during the malignant transformation from normal epithelium to low-grade intraepithelial neoplasia (LGIN) or high-grade intraepithelial neoplasia (HGIN). The expression of cystatin A and involucrin was significantly reduced from LGIN to HGIN. These results suggest c-Jun was involved in the regulation of differentiation-associated genes in ESCC. These genes might serve as the potential markers in distinguishing normal epithelium from esophageal squamous intraepithelial neoplasia.

## Introduction

Esophageal mucosa is lined by a stratified squamous epithelium that functions in cell proliferation, differentiation and turnover, and serves as a barrier [1]. Cell proliferation is frequent in the basal layers, and migration from this area eventually triggers differentiation [2]–[4]. Squamous cell differentiation requires the coordinated activation and repression of genes specific to the differentiation process, and disruption of normal differentiation accompanies neoplasia [5], [6]. The specific transcription factors including AP1, Ets1, KLF4 and Notch, control both of these processes and thereby play a pivotal role in the determination of cell fate [7]–[11]. Tissue-specific *KLF4* knockout mice had increased esophageal mucosa basal cell proliferation and a delay in cell maturation; these mice developed epithelial hypertrophy and subsequent dysplasia by 6 months of age [12]. Moreover, Ets1 disrupts the epidermal proliferation–differentiation equilibrium, and leads to severe dysplastic lesions in an inducible transgenic mouse model [13].

Transcription factor AP1, which mainly composed of Jun (c-Jun, JunB and JunD) and Fos (c-Fos, FosB and Fra-1), is an important modulator of cell function that control diverse biological processes [14]–[17]. Each AP1 complex subunit is expressed in a specific pattern in human epidermal, and can either induce or suppress gene expression [18]. c-Jun is the critical component of transcription factor AP1, and becomes transcriptionally active upon phosphorylation at Ser63 and Ser73 within its N-terminal transactivation domain by JNK [19], [20]. Exogenous expression of c-Jun promoted, whereas Jun B inhibited human epidermal neoplasia using the human SCC model regenerated on immune-deficient mice [21]. TAM67 is a dominant-negative form of c-Jun that interferes with all AP1 factors to inhibit transactivation [22]. AP1 factor inactivation by targeted expression of TAM67 in the suprabasal epidermis causes increased epidermal hyperproliferation and hyperkeratosis but reduced carcinogen-dependent tumor formation [23]–[25].

Our previous study showed that a group of genes involved in esophageal squamous cell differentiation were coordinately downregulated, including cystatin A, cytokeratins (KRT4, KRT13), small proline-rich proteins (SPRRs) and KLF4 [26]. These genes are all precursor proteins of cornified cell envelope (CE), and located in an evolutionarily conserved genetic cluster, designated as the epidermal differentiation complex (EDC) at the chromosome 1q21 [27], [28]. Several transcription factors including KLF4, Ets and AP1 were involved in expression of these genes during differentiation. Overexpression of KLF4 triggers the expression of SPRR1A, SPRR2A and KRT4 in esophageal cancer cell line KYSE70 [26]. Induction of Ets1 dramatically upregulated SPRRs expression, but involucrin was not changed using microarray analysis. Moreover, transcription factor AP1, a downstream effector of the Ras-MAPK pathway, is linked to the pathogenesis of ESCC. A common feature of the promoter regions of these genes is the presence of AP1 DNA binding sites, and regulated by TPA-induced c-Jun/AP1 mainly via a PKC/JNK pathway in esophageal cancer cell line KYSE450 [29].

Differentiation-associated genes cystatin A, involucrin, keratin 13 and SPRR3 are expressed in squamous epithelium during differentiation [30]–[32]. The processes of keratinocyte differentiation in esophagus have much in common with those in other squamous tissues, such as skin and oropharynx. In early stage of epidermal differentiation, expression of keratin proteins was upregulated, but in late stage of epidermal differentiation, the expression of CE proteins including involucrin, loricrin and SPRRs was upregulated [33]–[35]. Previous study showed that SPRR3 was down-regulated coordinately in ESCC, esophageal adenocarcinoma and anal cancer compared with corresponding normal epithelium [36], [37]. SPRR3 significantly suppressed cell proliferation and tumorigenicity of ESCC. However, the molecular mechanisms of transcription factor AP1 regulated differentiation- associated genes remain largely unknown.

In this study, we found that transcription factor c-Jun could bind to the promoters and activate the transcription of cystatin A, involucrin and SPRR3. Ectopic expression of c-Jun enhanced SPRR3 transactivation in KYSE450 cells. Conversely, TAM67, a dominant negative mutant of c-Jun, inhibited SPRR3 transactivation. In addition, SPRR3 was regulated by TPA-induced c-Jun mainly via a PKC/JNK pathway in KYSE450 cells. Moreover, we found that these genes were coordinately down-regulated, and associated with the differentiation grade in ESCC. Expression of c-Jun was closely correlated with the expression of these genes. The expression of cystatin A, involucrin and SPRR3 was remarkably decreased during the malignant transformation from normal epithelium to low-grade intraepithelial neoplasia (LGIN) or high-grade intraepithelial neoplasia (HGIN). From LGIN to HGIN, the expression of cystatin A and involucrin was significantly reduced. These findings demonstrated that these genes involved in the progression of ESCC were directly regulated by transcription factor c-Jun, and used as potential markers distinguishing normal epithelium from esophageal squamous intraepithelial neoplasia.

## Materials and Methods

### Cell line and cell culture

The ESCC cell lines (KYSE series) were kindly provided by Dr. Yutaka Shimada [38]. All ESCC cell lines used in this study were regularly authenticated by checking the morphology and were tested for the absence of mycoplasma contamination. All cells were maintained in RPM1640 supplemented with 10% fetal bovine serum, 100 U/ml penicillin and 100 µg/ml streptomycin at 37°C in a humidified 5% CO_2_ incubator.

### Reagent and antibodies

TPA was purchased from Sigma (Sigma-Aldrich, St. Louis, MO). PKC inhibitor (GF109203X), MEK1 inhibitor (PD98059) and JNK inhibitor (SP600125) were obtained from Calbiochem (Merck, Darmstadt, Germany). Antibodies were used as follows: c-Jun (Santa Cruz, Delaware, CA), cystatin A and β-actin (Sigma-Aldrich, St. Louis, MO), and involucrin (Lab Vision, Fremont, CA). SPRR3 antibody was made by our lab [36].

### Ethics statement

This study protocol had been reviewed and approved by the ethical committees of Chinese Academy of Medical Sciences Cancer Hospital. All participants gave written informed consent.

### Tissue specimens

110 paired ESCC tissue specimens (tumor and adjacent normal mucosa) and 30 precursor lesions (18 LGIN and 12 HGIN) were analyzed. None of the patients had received radiotherapy or chemotherapy before surgery. Clinical specimens were obtained at the time of surgery. The specimens were immediately fixed in 4% polyformaldehyde and completely embedded in paraffin. Clinical characteristics of the patients are summarized in Table 1.

**Table 1:** Patient characteristics.

Characteristics	Case no. (n = 110)	%
Age (years)		
≤60 years	57	52
>60 years	53	48
Sex		
Male	91	83
Female	19	17
Tumor location^a^		
Upper	8	7
Middle	68	62
Lower	32	29
Tumor size (cm)		
≤4 cm	24	22
>4 cm	86	76
Differentiation		
Well	24	22
Moderate-poor	84	76
Clinic stage		
I–II	81	74
III	26	24
LN metastasis		
Negative	75	68
Positive	35	32

Note: LN: lymph node;

a Position in the esophagus.

### Tissue microarray and immunohistochemistry

Tissue microarrays containing 110 paired tumor and adjacent normal mucosa (duplicate 0.6 mm tissue cores for each ESCC) were constructed. Sections (5 µm) were obtained from patients with precursor lesions. Informative expression of these differentiation-associated proteins was detected in TMA. Noninformative samples included lost sample and sample with too few tumor cells; such cases were excluded for data compilation. Immunohistochemistry staining was carried out following standard streptavidin-biotin-peroxidase complex method. Briefly, section was deparaffinized, and nonspecific bindings were blocked with 10% normal goat serum for 30 minutes. Section was then incubated with antibody overnight at 4°C. For negative controls, the primary antibody was replaced by non-immune serum. After immunostaining, the slides were examined independently by two pathologists blinded to both clinical and pathologic data.

Specimens were reviewed with staining intensity and staining extent. The intensity was graded as follows: 0, negative; 1, weak; 2, moderate; 3, strong. Staining extent was rated according to the percentage of positive cells in the field. The rate of positive cells was graded as follows: 0, <5%; 1, 5∼25%; 2, 26∼50%; 3, 51%∼75%; 4, >75%. Evaluation of the staining was achieved by multiplication of the extent of positivity and intensity. Scores of 0∼4 were defined as “negative expression”; scores of 4∼16 as “positive expression”.

After immunostaining, the sections from patients with precursor lesions were scan, and imaged by a single investigator who was not informed of the clinical characteristics. The value of the integral intensity was measured by Aperio's ImageScope software (Aperio,Vista, CA, USA).

### Plasmid construction and transfection

cystatin A, involucrin and SPRR3 promoter sequences were subcloned into the pGL3-basic vector (Promega, Madison, WI). pAP-1-luc and pCMV-myc-*c-Jun* were generous gifts from Dr. Lingqiang Zhang (Beijing Institute of Radiation Medicine, China) [39]. *TAM67* was amplified using the primers 5′-CAGCCAGAACACGCTGCCCAGCGTCACGTC-3′ and 5′- TCTTCGTTGCCCCTCAGCCCCCGACGGTCT-3′, then subcloned into pcDNA3.1 and further confirmed by sequencing. Transfections were done using Lipofectamine™ 2000 (Invitrogen, Carlsbad, CA) for plasmids according to the manufacturers' recommendations.

### Luciferase reporter assay

Cells were seeded in 96-well or 24-well plate, and transiently cotransfected with promoter constructs and *c-Jun* or *TAM67*. After 48 hours, luciferase activity was measured according to the manufacturer's protocol, and normalized for transfection efficiency by cotransfecting with 0.5 ng of pRL-SV40 Renilla (Promega).

### Chromatin immuneprecipitation assay (ChIP)

KYSE450 cells were cross-linked in 1% formaldehyde for 10 min at 37°C. DNA from the fixed-chromatin cells were then subjected to immunoprecipitation using ChIP assay kit with antibodies against c-Jun or β-actin (Upstate, Lake Placid, NY). Purified DNA was analyzed by PCR with the primers: cystatin A, 5′-GGGGCTTCCTCCATATCTG-3′ and 5′-AGGATGAACAAGTG GGCAAG-3′; involucrin, 5′-AGCAGCAGAGGACTCCTAG-3′ and 5′-GTGATGGACAGG TTT CACC-3′; and SPRR3, 5′-GCCTGGCTCGTTCTAGTC-3′ and 5′-AGGTGTTCCT AATGCGGAG-3′. PCR product was resolved on a 2% agarose gel.

### Western blot

Western blot was done according to the standard protocol with antibodies c-Jun, involucrin, cystatin A and SPRR3. β-actin was used as loading control. Western blots expression data was quantified using ImageJ.

### Statistical analysis

Statistical analysis was performed using two-tailed, two-independent or paired sample *t* tests, one-way ANOVA tests depending on the number of groups with SPSS17.0 (SPSS, Chicago, IL). Relationship between the expression of the genes and the clinical pathologic characteristics was analyzed using a χ^2^ test. The correlation was analyzed using Pearson correlation analysis. P < 0.05 was considered significant.

## Results

### c-Jun could transactivate differentiation-associated genes

**Figure 1:**
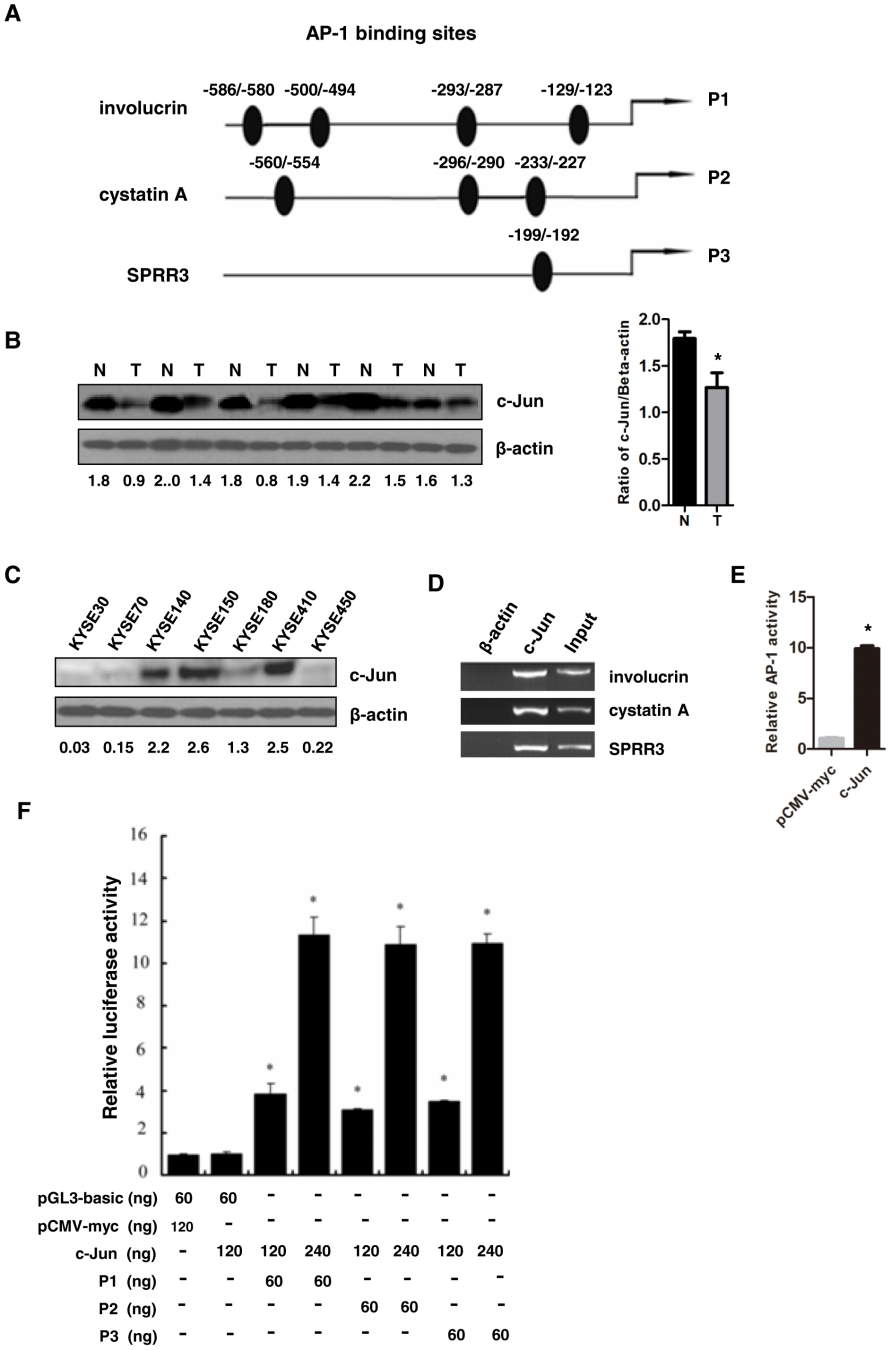
c-Jun could transactivate differentiation-associated genes. (**A**) A schematic illustration of human cystatin A, involucrin and SPRR3 genes. AP-1 DNA binding sites predicted using bioinformatics software. (**B**) Down-regulation of c-Jun was frequently detected in the paired ESCC tissues (T) and adjacent nontumor tissues (N) from the same patient by Western blot (left panel). Statistical analysis was performed (right panel). (**C**) Expression of c-Jun was detected in esophageal cancer cell lines by Western blot. (**D**) c-Jun can directly bind to the promoters of these genes in vivo. KYSE450 cells were subjected to ChIP with anti-c-Jun or β-actin antibody. Purified DNA was analyzed by PCR. (**E**) AP-1 transactivation activity was detected by luciferase reporter assay system. KYSE450 cells were transiently cotransfected with pAP-1-luc and pRL-CMV. (**F**) c-Jun could transactivate differentiation- associated genes. KYSE450 cells were cotransfected with c-Jun and cystatin A, involucrin or SPRR3 promoter constructs. pRL-SV40 Renilla was used for normalization of transfection efficiency. After 48 hours, the luciferase activity was measured. Data shown are Mean ± SD from multiple independent experiments (*: P< 0.05).

Our previous study indicated that a group of differentiation-associated genes were coordinately down-regulated in human ESCC [26]. A common feature of the promoter regions of these genes, including cystatin A, involucrin and SPRR3, is the presence of AP1 DNA-binding sites (Figure 1A). To assess whether c-Jun directly regulated these genes, we first examined c-Jun status in ESCC. As shown in Figure 1B, c-Jun was reduced in 8 of 13 paired ESCC tissues detected by Western blot. Next, we found that the expression level of c-Jun was low in esophageal cancer cell lines KYSE30, KYSE70, KYSE180 and KYSE450, but high in KYSE140, KYSE150 and KYSE410 (Figure 1C). As shown in Figure 1D, c-Jun could bind to the promoters of these genes *in vivo* in KYSE450 cells by chromatin immunoprecipitation (ChIP) assays, which is in agreement with previous report that c-Jun binds to the region of the cystatin A and involucrin promoter [40], [41]. To test whether AP1 DNA-binding activity had functional consequences, we performed transient transfections with pAP-1-luc and pCMV-c-Jun into KYSE450 cells to analyze AP-1 transactivation activity. c-Jun obviously increased AP1 activity (Figure 1E). Thus, we performed luciferase report assay by transient cotransfection into KYSE450 cells with pCMV-c-Jun and various promoter constructs. As shown in Figure 1F, c-Jun increased the promoter activities of cystatin A, involucrin, and SPRR3 in a dose-dependent manner, suggesting that c-Jun activates the promoters of these differentiation-associated genes *in vivo* in esophageal cancer cell line.

### TAM67, a dominant-negative form of c-Jun suppressed SPRR3 expression

**Figure 2:**
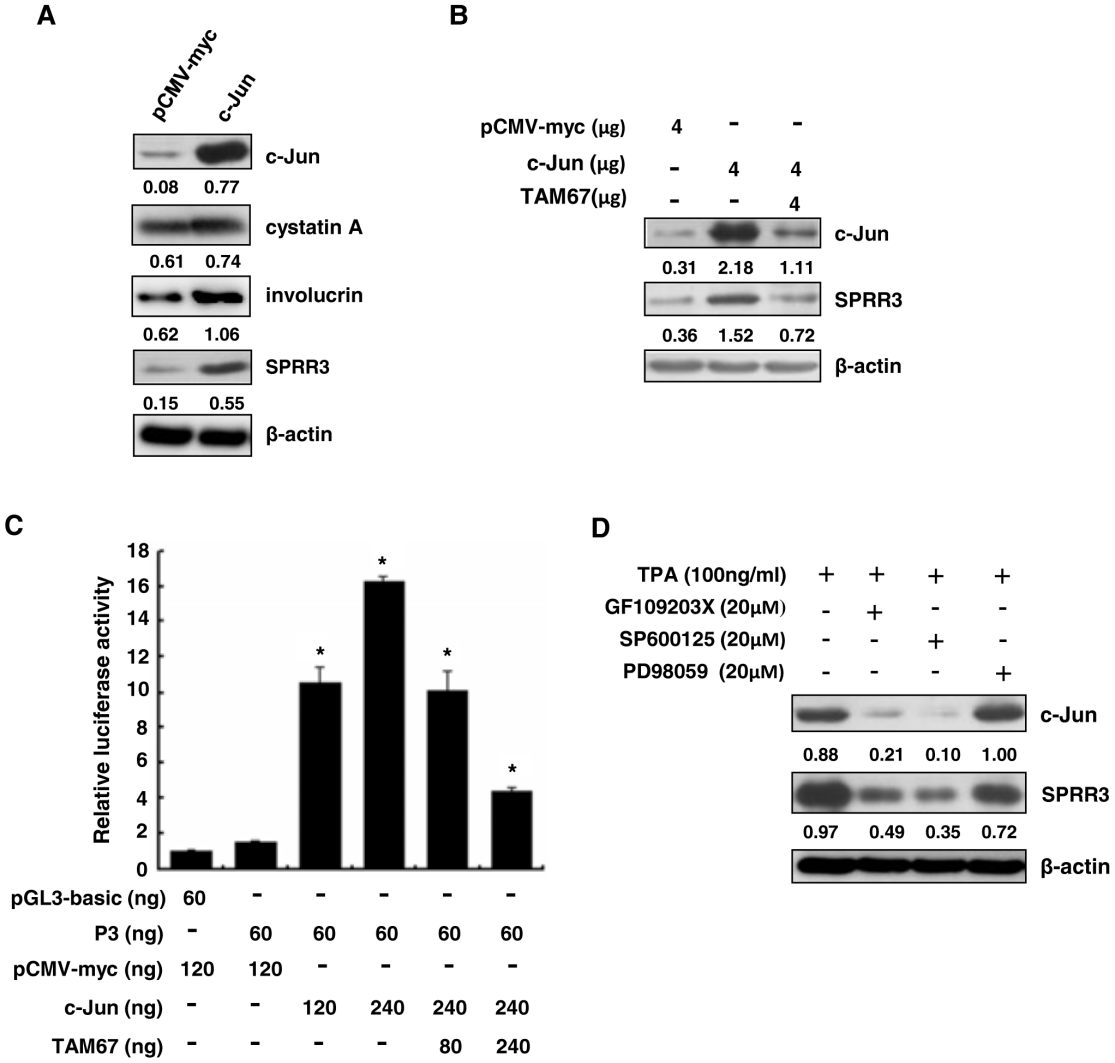
c-Jun promoted expression of SPRR3, but TAM67 suppressed expression of SPRR3. (**A**) KYSE450 cells were transiently transfected with pCMV-c-Jun. After 48 hours, cells were lysed, and analyzed by Western blot. (**B**) KYSE450 cells were transfected with pCMV-c-Jun and (or) pcDNA3.1-TAM67. After 48 hours, cells were lysed, and detected by Western blot. (**C**) TAM67 suppresses AP1 factor-dependent promoter activity. KYSE450 cells were cotransfected with the promoter construct of SPRR3 and pCMV-c-Jun or (and) pcDNA3.1-TAM67. pRL-SV40 Renilla was used for normalization of transfection. Data shown are Mean ± SD from multiple independent experiments (*: P< 0.05). (**D**) MAPK inhibitors suppressed TPA-triggered activation of c-Jun. KYSE450 cells were pretreated with 20 µM PKC inhibitor (GF109203X), JNK inhibitor (SP600125) or MEK inhibitor (PD98059) for 1 hour respectively, and then treated with 100 ng/ml TPA for 8 hours. Cells were lysed. The expression levels of c-Jun and SPRR3 were examined by Western blot. β-actin was used as a loading control.

As shown above, c-Jun can transcriptionally activate the promoters of differentiation-associated genes, thus we further examined protein levels of these genes by transient transfection of c-Jun. We found that ectopic c-Jun remarkably increased the expression of SPRR3 in KYSE450 cells (Figure 2A). Consistent with the previous study, we also found that expression of cystatin A and involucrin was activated by overexpression of c-Jun. Thus, we mainly focus on the regulation mechanism of SPRR3. TAM67 is a dominant-negative form of c-Jun that interacts broadly with all AP1 transcription factors to inhibit transactivation [22]. As shown in Figure 2B, ectopic c-Jun increased expression of SPRR3, whereas TAM67 reduced expression of SPRR3 in KYSE450 cells. To assess the molecular mechanism, we monitored the impact on SPRR3 promoter activity. As shown in Figure 2C, SPRR3 promoter activity was inhibited by TAM67 using luciferase report assay. These results indicated that TAM67 suppress differentiation-associated AP1-dependent transcriptional events in esophageal cancer cells.

### SPRR3 was regulated by TPA-induced c-Jun expression via a PKC/JNK pathway

Our previous study showed that c-Jun/AP1 DNA-binding activity and transactivation activity are mediated by 12-O-tetradecanoylphorbol-13-acetate (TPA) in KYSE450 cells [29]. To further investigate whether the expression of c-Jun was regulated by MAPK pathway, we utilized the specific pharmacological inhibitors of PKC (GF109203X), JNK (SP600125) and MEK (PD98059) respectively to prevent MAPKs in response to TPA stimulation. Indeed, GF109203X and SP600125 prominently inhibited the expression of SPRR3, whereas PD98059 had little effect in KYSE450 cells (Figure 2D). These findings suggest that c-Jun promotes expression of SPRR3 mainly via a PKC/JNK pathway.

### c-Jun and differentiation-associated genes were coordinately down-regulated in ESCC

**Figure 3:**
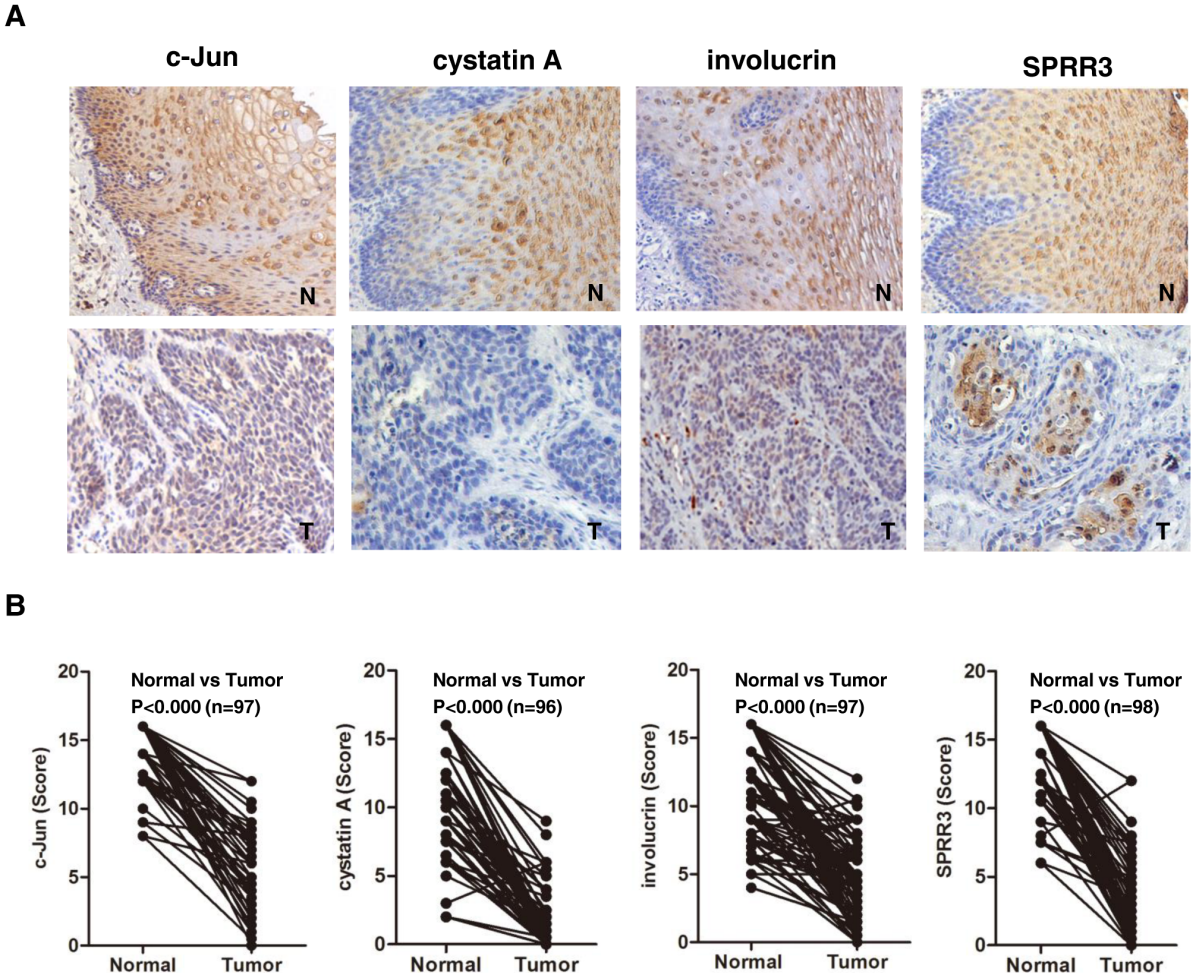
c-Jun and differentiation-associated genes were coordinately down-regulated in ESCC. (**A**) Expression of c-Jun, cystatin A, involucrin or SPRR3 was examined in human ESCC using TMA on 110 pairs of ESCCs and their corresponding nontumor tissues by immunohistochemistry. (**B**) Statistical analysis was performed using SPSS17.0 software. c-Jun and differentiation-associated genes were significantly down-regulated in ESCC.

As shown above, c-Jun directly regulated the expression of differentiation-associated genes in esophageal cancer cell line, thus we further determine the protein levels of these genes in ESCC tissues using immunohistochemistry. As shown in Figure 3A, c-Jun was expressed in the intermidated and superficial layer of normal epithelium, and displayed cytoplasmic and nuclei localization, whereas it was predominantly localized in nuclei in the tumor tissues with more diffuse location. A similar pattern of cystatin A, involucrin and SPRR3 immunoreactivity was observed. These proteins were coordinately down-regulated in ESCC tissues compared with the adjacent normal mucosa. In normal epithelium, cystatin A, involucrin and SPRR3 was predominantly expressed in cytoplasm in intermediate and superficial layers, whereas they were weakly or not expressed in basal layer. We found that the expression level of these proteins was significantly reduced in tumor tissues compared with the normal counterparts (Figure 3B). These results showed that the expression of c-Jun and differentiation-associated genes were coordinately down-regulated, and these genes may play a role in the pathogenesis of esophageal neoplasia. The above result prompted us to analyze whether a direct relationship exists between c-Jun and differentiation-associated genes. We found that there was also a statistical correlation between expression of c-Jun and these proteins in ESCC and normal epithelium (Table 2). In normal epithelium, c-Jun correlated with cystatin A (P<0.001), involucrin (P = 0.003) or SPRR3 (P<0.001), and in ESCC, c-Jun significantly correlated with cystatin A (P<0.001), involucrin (P = 0.021) and SPRR3 (P = 0.019) as well. Thus, we speculate that transcription factor c-Jun may participate in the process of altering expression of differentiation-associated genes in ESCC.

**Table 2:** Correlation between c-Jun with differentiation-associated genes.

	Normal epithelium		ESCC	
	(n = 98)		(n = 108)	
	R	P	R	P
c-Jun and cystatin A	0.429	0.000	0.353	0.000
c-Jun and involucrin	0.302	0.003	0.224	0.021
c-Jun and SPRR3	0.476	0.000	0.226	0.019

Note: R value is pearson′s correlation coefficient; P<0.05 is considered as significant.

### Association between the expression of differentiation-associated genes and clinico- pathologic characteristics

**Figure 4:**
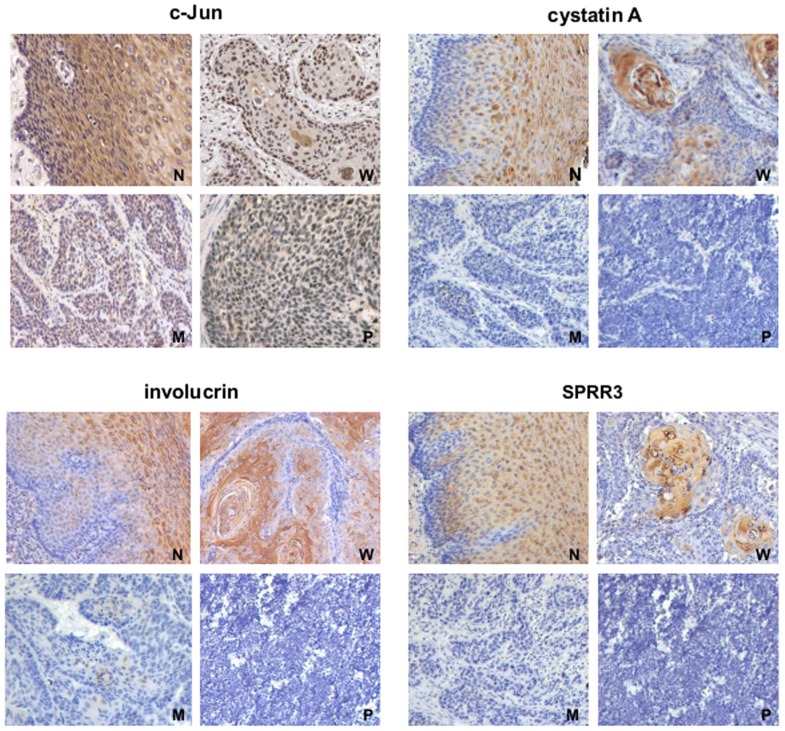
Expression of differentiation-associated genes is associated with differentiation stage in ESCC. Immunohistochemistry showed expression pattern of c-Jun and differentiation-associated genes in ESCC. A similar pattern of c-jun, cystatin A, involucrin and SPRR3 immunoreactivity was observed. These proteins were highly expressed in the intermediate and superficial layers in normal epithelium. c-Jun was displayed both cytoplasmic and nuclei localization in normal epithelium, whereas it was located in the cytoplasm in the tumor nest. cystatin A, involucrin and SPRR3 were predominantly localized in well-differentiation ESCC, but weakly expressed in moderately and poorly differentiated ESCC. N: nontumor tissue; W: well-differentiated ESCC; M: moderately differentiated ESCC; P: poorly differentiated ESCC.

To further investigate correlation between the expression of differentiation-associated genes and the clinicopathological parameters in ESCC, we then performed statistical analysis. As shown in Figure 4, the strong staining of cystatin A, involucrin and SPRR3 was predominantly localized in the hyperkeratotic portion of tumor nests in well-differentiated ESCC tissues, but there was weak or negative staining in moderately- or poorly-differentiated ESCC tissues. cystatin A, involucrin and SPRR3 expression was significantly higher in carcinoma with well differentiation than that with moderate or poor differentiation (P = 0.002, P = 0.003 and P = 0.010, respectively). However, c-Jun expression has no relationship with the differentiation grade (Table 3). Moreover, we found that the expression of involucrin and SPRR3 was also correlated with the clinical stage (P = 0.033 and P = 0.030, respectively). These data suggested that cystatin A, involucrin and SPRR3 might be used as potential differentiation markers of esophageal epithelium.

**Table 3:** Correlation of immunohistochemistry abnormalities with clinicopathologic characteristics in ESCC.

		Sex		Differentiation		Clinic stage		LN metastasis	
Protein		Male	Female	W	M-P	I–II	III	−	+
Case No		90	18	30	78	80	25	74	34
c-Jun	+	21	2	2	18	15	8	14	9
	++	69	16	21	60	65	17	60	25
	P	0.248		0.098		0.162		0.373	
cystatin A	+	81	13	14	73	69	22	65	29
	++	9	5	10	10	11	3	9	5
	P	0.040*		0.002*		0.822		0.715	
involucrin	+	36	4	2	34	25	14	23	17
	++	54	14	22	50	55	11	51	17
	P	0.154		0.003*		0.033*		0.059	
SPRR3	+	60	10	7	51	47	22	44	26
	++	30	8	17	33	33	3	30	8
	P	0.422		0.010*		0.030*		0.128	

Note: W: Well-differentiated ESCC; M: Moderately differentiated ESCC; P: Poorly differentiated

ESCC; LN: Lymph node; +, Low expression; ++, High expression; *P<0.05 is considered as significant.

### c-Jun, cystatin A, involucrin and SPRR3 involved in intraepithelial neoplasia

**Figure 5:**
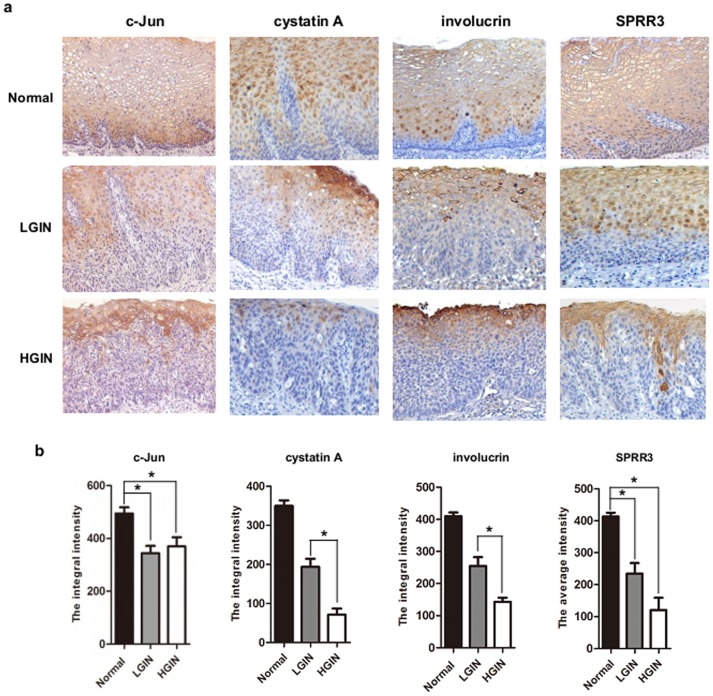
Representative photographs of immunohistochemical features of these proteins in different lesions. (**A**) Expression of c-Jun, cystatin A, involucrin and SPRR3 were detected in the precursor lesions, including normal epithelium, low-grade intraepithelial neoplasia (LGIN) and high-grade intraepithelial neoplasia (HGIN) using immunohistochemistry. From the normal epithelium to LGIN and HGIN, expression of these genes decreased markedly and sometimes even totally disappeared in LGIN and HGIN. (**B**) Statistical analysis was performed to determine the expression of these proteins during the progress of esophageal tumorigenesis using SPSS17.0 software.

Squamous differentiation requires the coordinated activation and repression of genes specific to the differentiation process, and disruption of this program accompanies neoplasia. To determine whether these genes were involved in the pathogenesis of epithelium neoplasia, we detected the expression of these proteins using immunohistochemistry in precursor lesions including normal epithelium, LGIN and HGIN. As shown in Figure 5A, c-Jun showed strong staining in intermediate and superficial layers except for the basal cell layer in normal epithelium. Such difference in the staining pattern and intensity allowed us to easily pinpoint the margin between dysplastic and normal squamous epithelium. With advanced degree of dysplasia from normal epithelium to LGIN or HGIN, expression of c-Jun was remarkably decreased. Interestingly, expression of these proteins in the precursor lesions was compatible with that of c-Jun. cystatin A, involucrin and SPRR3, which are normally expressed exclusively in the basal layer, are detected in all epidermal layers. As shown in Table 4, the expression of these proteins in normal epithelium was significantly higher than the preceding precursor lesion. Moreover, we found that the expression of cystatin A and involucrin was significantly reduced from LGIN to HGIN (Figure 5B). These results indicated that dysfunction of c-Jun and differentiation-associated genes were very early event in the progress of esophageal squamous intraepithelial neoplasia.

**Table 4:** Immunohistochemistry abnormalities in different lesions of ESCC.

		Precursor lesions		*P*-value		
Protein	Normal	LGIN	HGIN	*P^a^*	*P^b^*	*P^c^*
	Mean ± SD	Mean ± SD	Mean ± SD			
c-Jun	494.3±88.4	344.4±77.5	369.6±114.7	0.000	0.005	0.598
cystatin A	349.7±41.9	193.9±46.3	71.48±45.9	0.000	0.000	0.000
involucrin	409.6±38.7	254.9±82.7	142.9±25.9	0.000	0.000	0.025
SPRR3	413.3±43.5	234.8±108.8	120.6±76.7	0.000	0.000	0.078

Note: Mean: Average of the integral intensity of all samples; a, Esophageal normal epithelium vs LGIN; b, Esophageal normal epithelium vs HGIN; c: LGIN vs HGIN.

## Discussion

Esophageal basal cells are subjected to a tightly regulated program of differentiation as they migrate toward the surface accompanied by a series of morphological, biochemical and genetic changes [42], [43]. Squamous differentiation requires the coordinated activation and repression of genes specific to the differentiation process, and disruption of this program accompanies neoplasia [6], but the genetic mechanisms of this process are still poorly understood. In this study, we found that cystatin A, involucrin and SPRR3 were dramatically down-regulated, and significantly correlated with differentiation grade in ESCC. These genes expressed differently in basal versus differentiation layer. Previous study showed that involucrin was specifically found in the suprabasal but not basal keratinocytes of the esophagus [8]. However, we found that cystatin A, involucrin and SPRR3 were strongly expressed in the intermediated and superficial layer of normal epithelium, whereas disappeared in basal layer. Moreover, cystatin A, involucrin and SPRR3 are all the precursor proteins of cornified cell envelope [32], [44], [45]. Knockouts of the major CE constituent loricrin or involucrin, envoplakin and periplakin significantly disrupt the barrier function of the skin [46], [47]. Thus, dysfunction of these genes might lead to the disability of the formation of CE, and disrupts proliferation and differentiation of esophageal epithelium. Our results suggested that cystatin A, involucrin and SPRR3 might serve as the potential differentiation markers of esophageal epithelium.

The metaplasia → dysplasia → carcinoma sequence is characterized by an increased proliferation rate, balance of differentiation, expression of specific genes and abnormally increased apoptosis [48]. Dysplasia especially HGIN remains the most predictive factor for ESCC. Previous study showed that cyclin D1 expression and hypermethlaytion or mutation of p16 are the critical early events. Xue *et al*. showed that overexpression of fascin, FADD, CDC25B and underexpression of CK4, annexin I, Fas, caspase 8 are also the early events. Overexpression of CK14, laminin-5γ2 and SPARC may be later events in this sequence [49]. Recently, epidemiologic studies suggest only HGIN were significantly associated with increased risk of developing ESCC. The progression to cancer in patients with HGIN was observed to be up to 34%. Early detection offers the best prognosis for ESCC. Wang *et al*. showed that Ki67 and ProExC exhibited high sensitivity and specificity for distinguishing reactive hyperplasia from LGIN and HGIN [50]. In this study, we found that c-Jun, cystatin A, involucrin and SPRR3 expressed exclusively in the basal layer, are detected in all epidermal layers, but low in LGIN and HGIN. From LGIN to HGIN, the expression of cystatin A and involucrin was significantly reduced. These results suggest that c-Jun and these proteins might serve as markers distinguishing normal/reactive hyperplasia from esophageal squamous intraepithelial neoplasia.

Previous study showed that several transcription factors were involved in epithelium cell proliferation and differentiation [12], [13]. A function for AP-1 activity in skin tumorigenesis has been identified using knockout and transgenic mice that modulate AP-1 components. Mice lacking *c-Jun* die at mid-gestation and their embryonic lethality are associated with increased apoptosis in fetal liver cells [51]–[53]. A transactivation mutant of c-Jun (TAM67) expressed under the Keratin 14 promoter or an inducible system produces a thickened, hyperproliferative, parakeratotic epidermis, but the epidermis is resistant to DMBA/TPA-dependent tumor formation [21], [24], [25], [54]. Consistent with JunB expression status in squamous cell carcinoma [21], c-Jun is remarkably reduced in human ESCC. Staining of c-Jun was strong in differentiation layers, and disappeared in the basal layer. With the progression of carcinogenesis, expression of c-Jun was significantly decreased. These data suggest that c-Jun, regulates differentiation-associated genes, and inhibits esophageal epithelium neoplasia.

Transcription factor c-Jun contributed to esophageal epithelial homeostasis, and regulated the program of differentiation. A common feature of the promoter regions of these genes, including cystatin A, involucrin and SPRR3, is the presence of AP-1 DNA-binding sites [40], [41], [44]. Our study indicated c-Jun can directly bind to the AP-1 binding sites in promoter regions of cystatin A, involucrin and SPRR3 in vivo, and activated the transcription of these genes. Previous study showed that KRT13, an esophageal-specific cytokeratin, implies tissue-specific differentiation [55]. We also found that c-Jun transactivated KRT13 in our model (data not shown). Ectopic expression of c-Jun increased SPRR3 expression in KYSE450 cells, whereas TAM67, a dominant-negative mutant of c-Jun, can significantly block c-Jun induced promoter activity and SPRR3 expression. Moreover, c-Jun up-regulated SPRR3 expression induced by TPA via a PKC/JNK pathway in KYSE450 cells. These results indicate that c-Jun is a critical factor for expression of SPRR3 in esophageal cancer cell.

## Conclusion

In summary, down-regulation of c-Jun may disrupt squamous cell differentiation, and lead to basal cell hyperplasia and dysplasia in the esophagus. cystatin A, involucrin and SPRR3 might be served as the potential differentiation markers of ESCC, and used as potential markers distinguishing normal epithelium from esophageal squamous intraepithelial neoplasia.
